# Prognostic Significance of Overexpressed p16^INK4a^ in Patients with Cervical Cancer: A Meta-Analysis

**DOI:** 10.1371/journal.pone.0106384

**Published:** 2014-09-04

**Authors:** Jiaying Lin, Andreas E. Albers, Jinbao Qin, Andreas M. Kaufmann

**Affiliations:** 1 Clinic for Gynecology, Charité-Universitätsmedizin Berlin, Berlin, Germany; 2 Department of Otolaryngology, Head and Neck Surgery, Charité-Universitätsmedizin Berlin, Berlin, Germany; 3 Department of Vascular Surgery, Shanghai Ninth People’s Hospital Affiliated to Shanghai JiaoTong University School of Medicine, Shanghai, P. R. China; National Institute of Health - National Cancer Institute, United States of America

## Abstract

**Background:**

p16^INK4a^ is a tumor suppressor protein which is induced in cells upon the interaction of high-risk HPV E7 with the retinoblastoma protein by a positive feedback loop, but cannot exert its suppressing effect. Previous reports suggested that p16^INK4a^ immunostaining allows precise identification of even small CIN or cervical cancer lesions in biopsies. The prognostic value of overexpressed p16^INK4a^ in cervical cancer has been evaluated for several years while the results remain controversial. We performed a systematic review and meta-analysis of studies assessing the clinical and prognostic significance of overexpression of p16^INK4a^ in cervical cancer.

**Methods:**

Identification and review of publications assessing clinical or prognostic significance of p16^INK4a^ overexpression in cervical cancer until March 1, 2014. A meta-analysis was performed to clarify the association between p16^INK4a^ overexpression and clinical outcomes.

**Results:**

A total of 15 publications met the criteria and comprised 1633 cases. Analysis of these data showed that p16^INK4a^ overexpression was not significantly associated with tumor TNM staging (I+II vs. III+IV) (OR = 0.75, 95% confidence interval [CI]: 0.35–1.63, P = 0.47), the tumor grade (G1+ G2 vs. G3) (OR = 0.78, 95% CI: 0.39–1.57, P = 0.49), the tumor size (<4 vs. ≥4 cm) (OR = 1.10, 95% CI: 0.45–2.69, P = 0.83), or vascular invasion (OR = 1.20, 95% CI: 0.69–2.08, P = 0.52). However, in the identified studies, overexpression of p16^INK4a^ was highly correlated with no lymph node metastasis (OR = 0.51, 95% CI: 0.28–0.95, P = 0.04), increased overall survival (relative risk [RR]: 0.42, 95% CI: 0.24–0.72, P = 0.002) and increased disease free survival (RR: 0.60, 95% CI: 0.44–0.82, P = 0.001).

**Conclusions:**

This meta-analysis shows overexpression of p16^INK4a^ in cervical cancer is connected with increased overall and disease free survival and thus marks a better prognosis.

## Introduction

Cervical cancer is the third most common malignancy in women worldwide and a leading cause of cancer-related death in women in developing countries [1]. The relationship between the development of cervical cancer and persistent infection with high-risk human papilloma virus (HR-HPV) is well established [2]. A plethora of research on the development of objective biomarkers allowing to distinguish the transformation from productive HPV infections to carcinoma and to predict disease severity has been performed. The cellular tumor suppressor protein cyclin-dependent kinase inhibitor 2A (p16^INK4a^) has been identified as a biomarker for transforming HPV infections [3]. It decelerates the cell cycle by inactivating the cyclin-dependent kinases (CDK4/CDK6) involved in the phosphorylation of the retinoblastoma protein (pRb) [4]. This process is leading to senescence in normal cells. In the presence of the HR-HPV oncogene E7, p16^INK4a^ transcription is induced by the histone demethylase KDM6B [5]. HPV E7 expression causes an acute dependence on KDM6B expression for cervical cancer cell survival. Thus, the p16^INK4A^ tumor suppressor is a critical KDM6B downstream transcriptional target and its expression is critical for cell survival [6].

Over the past decade, several studies have evaluated the prognostic value of p16^INK4a^ protein expression in cervical cancer with conflicting results. Some concluded that p16^INK4a^ expression had no influence on survival [7] while others reported that p16^INK4a^ expression was predictive of improved survival outcome for cervical carcinoma [8], [9]. In order to evaluate this question, we conducted a systematic review and meta-analysis to determine the association between the overexpression of p16^INK4a^ and common clinical and pathologic features of cervical cancer.

## Materials and Methods

### Search strategy

A comprehensive literature search of the electronic databases PubMed (www.pubmed.com), EMBASE (www.embase.com) and Wanfang (www.wanfangdata.com.cn) was performed up to March 1, 2014. The following search terms and their combinations were used: “cervical cancer,” “cervical carcinoma,” “carcinoma of cervix”, “CDKN2A”, “p16” and “p16^INK4a^”. The citation lists associated with all the studies retrieved in the search were used to identify other potentially relevant publications. Review articles were also scanned to find additional eligible studies but none could be identified from the reference lists. The title and abstract of each study identified in the search was scanned to exclude any clearly irrelevant publications. The remaining articles were browsed to determine whether they contained information on the topic of interest.

### Selection criteria

Diagnosis of cervical cancer was proven by histopathological methods. Studies of p16^INK4a^ expression based on cervical cancer tissue (after either surgical excision or biopsy sampling) were included. Studies based on serum or any other kinds of specimen were excluded. All studies on the correlation of p16^INK4a^ overexpression with clinicopathological markers and the association of p16^INK4a^ overexpression on overall survival (OS) or disease-free survival (DFS) of cervical cancer patients were included. For inclusion into the analysis, there was no limitation on the minimum number of patients of every single study. When there were multiple articles by the same group based on similar patients and using same detection methods, only the largest or the most recent article was included.

### Data extraction

Information was carefully extracted from all eligible publications independently by two of the authors according to the inclusion criteria listed above. Disagreement was resolved by a consensus discussion between the two authors. Data tables were composed to extract all relevant data from texts, tables, and figures of each included study, including author, publication year, country of patient’s origin, tumor stage, number of patients, research technique used, and cutoff value of overexpression of p16^INK4a^. In case the prognosis was only plotted as Kaplan-Meier curve in some articles, the software GetData Graph Digitizer 2.24 (free software downloaded at http://getdata- graph-digitizer.com) was applied to digitize and extract the data.

### Statistical analysis

The cut-off for p16^INK4a^ –positivity according to stained cells is given in table 1 for every study included. ORs with 95% confidence intervals (CI) were used to evaluate the association between p16^INK4a^ overexpression and clinicopathological factors, including tumor TMN staging, tumor grade, tumor size, vascular invasion and lymph node status. To stratify for the analysis, the following data of p16^INK4a^ overexpression and clinicopathological factors were combined into single categories with comparable clinicopathologic relevance: tumor TMN staging (I+II vs. III+IV); tumor grade (G1+G2 vs. G3); tumor size (<4 vs. ≥4 cm); vascular invasion or not; lymph node negative or positive.

**Table 1:** Main characteristics and results of the eligible studies.

Study	Patient’s country	Year	Tumor stage(UICC)	Technique	Percentage of p16^INK4a^ positive cells	Number of patients	Cut off (IHC)
Li et al.	China	2000	ND	IHC	54%	72	ND
Alfsen et al.	Norway	2003	I-II	IHC	73%	138	>50%
Van de Putte et al.	Norway	2003	ND	IHC	43%	212	>50%
Han et al.	China	2004	I–IV	IHC	42%	43	>50%
Masoudi et al.	Canada	2006	ND	IHC	83%	130	ND
Huang et al.	China	2007	I–IV	IHC	65%	89	>1%
Cao et al.	China	2008	I-III	IHC	24%	41	>25%
Bodner et al.	Austria	2011	I–IV	IHC	56%	39	>1%
Huang et al.	Taiwan	2011	I–IV	IHC	74%	145	staining scores>2
Schwarz et al.	USA	2011	I–IV	IHC	94%	126	>1%
Mu et al.	China	2012	I-III	IHC	63%	275	ND
Jiang et al.	China	2012	I-II	IHC	79%	76	>5%
Weng et al.	China	2012	I–IV	IHC	61%	62	>10%
Son et al.	Korea	2012	ND	IHC	74%	35	>5%
Liu et al.	China	2012	I–IV	IHC	83%	150	>5%

IHC; immunohistochemistry; ND: not documented.

The RR with 95% CI was used for assessing the association of p16^INK4a^ and the survival outcome combined over studies. An observed OR or RR <1 implied a better prognosis for the group with p16^INK4a^ overexpression and would be considered to be statistically significant if the 95% CI did not exceed 1. The existence of heterogeneity between studies was evaluated using the Dersimonian and Laird’s Q test [10]. *I* ^2^ was used to quantify heterogeneity and an *I* ^2^ value >50% was considered to represent substantial heterogeneity between studies. Relative to fixed-effects models, random-effects models were used and were more appropriate for the current study, because of the heterogeneity visible from the forest plots which often cannot be revealed by the Q test given its low power. The influence of individual studies on the summary effect estimate was displayed using the sensitivity analysis. In addition, funnel plots and the Egger’s test were used to estimate the possible publication bias [11]. Cochrane Review Manager, version 5.2 (Cochrane Library, Oxford, UK) was used to calculate the ORs or RRs and their variations from each investigation.

## Results

### Description of studies

**Figure 1:**
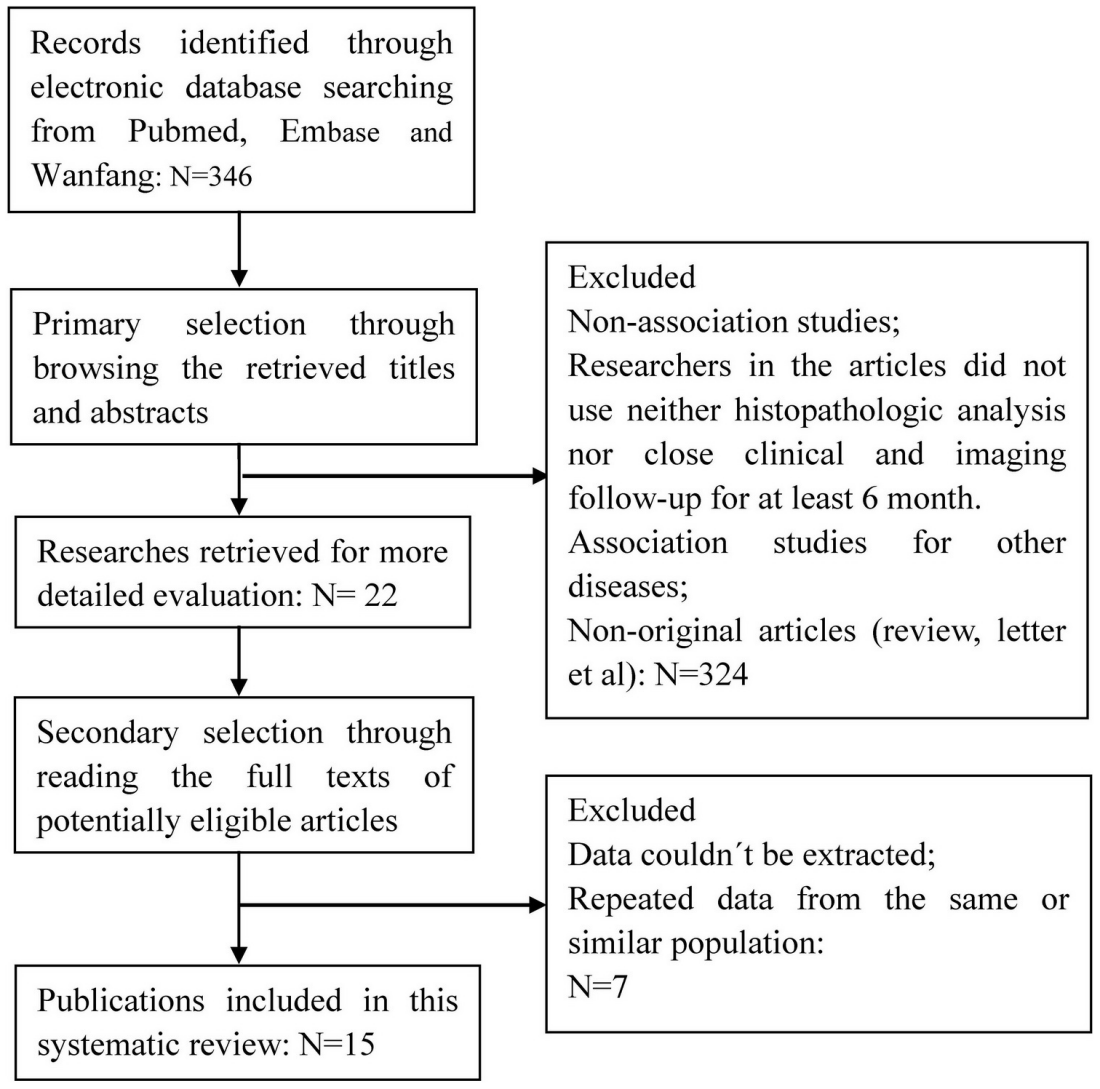
Literature search strategy and selection of articles. A total of 346 articles were selected for the meta-analysis by browsing the databases PubMed, Embase and Wanfang, of which 324 were excluded after reviewing the title and abstract, seven articles were excluded after reviewing the full publications, the reasons for exclusion were: (a) Non-association studies (b) researchers in the article did not use neither histopathologic analysis nor close clinical and imaging follow-up for at least 6 months, (c) association studies for other diseases (d), non-original articles, (e) data couldn’t be extracted or (f) repeated data from the same or similar population. Finally, a total of 15 studies with 1633 patients, who fulfilled all of the inclusion criteria, were considered for the analysis.

A total of 15 publications met the criteria for this analysis [7]–[9], [12]–[23] (Fig. 1). The total number of patients was 1633, ranging from 35 to 275 patients per study. Main characteristics of the eligible studies are summarized in Table 1 including the cut-off definition for p16^ INK4a^ positivity. Thirteen articles dealt with clinicopathological factors. Eight studies determined overall survival (OS) or disease free survival (DFS). Seven studies only reported the association between p16^INK4a^ overexpression and clinicopathological factors without OS or DFS analysis. There was only one method used to evaluate p16^INK4a^ expression in cervical cancer specimens i.e. immunohistochemistry (IHC).

### Correlation of p16^INK4a^ expression with clinicopathological parameters

**Figure 2:**
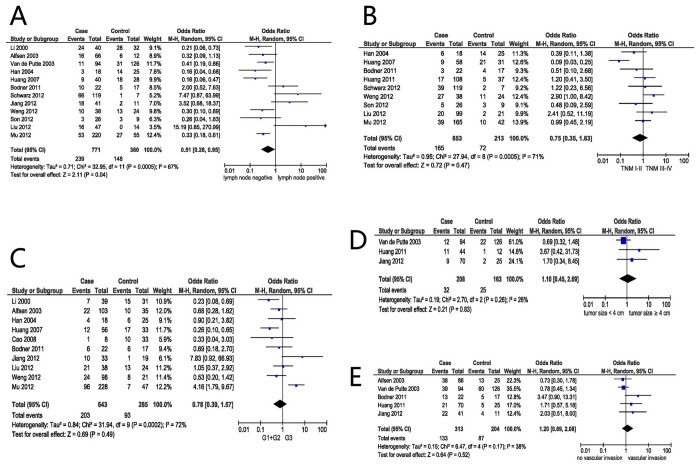
Forest plot depiction of p16^INK4a^ expression and OR for clinical pathologic features. Clinicopathological parameters investigated are lymph node status (A), TMN classification (B), tumor grade (C), size of tumor (D), vascular invasion (E). OR with corresponding confidence intervals are shown.

The association between p16^INK4a^ and several clinicopathological parameters is illustrated in Figure 2. Overexpression of p16^INK4a^ was highly correlated with no lymph node metastasis (OR = 0.51, 95% CI: 0.28–0.95, P = 0.04) (Fig. 2A). However, overexpression of p16^INK4a^ was not significantly associated with tumor TNM staging (I+II vs. III+IV) (OR = 0.75, 95% CI: 0.35–1.63, P = 0.47) (Fig. 2B), the tumor grade (G1+ G2 vs. G3) (OR = 0.78, 95% CI: 0.39–1.57, P = 0.49)(Fig. 2C), the tumor size (<4 vs. ≥4 cm) (OR = 1.10, 95% CI: 0.45–2.69, P = 0.83) (Fig. 2D), or vascular invasion (OR = 1.20, 95% CI: 0.69–2.08, P = 0.52) (Fig. 2E).

### p16^INK4a^ expression and 5-year survival outcome

**Figure 3:**
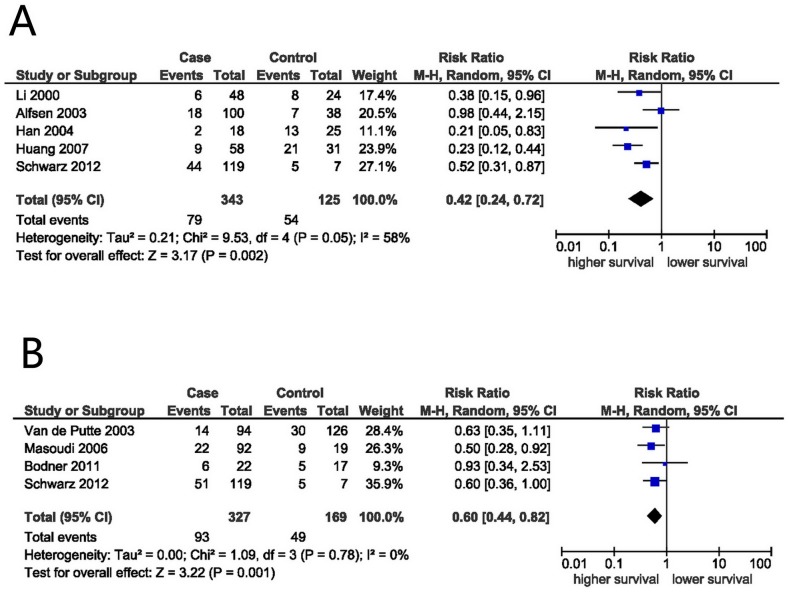
Analysis of p16^INK4a^ expression and survival of cervical cancer patients. Forest plot of RR for OS (A) and DFS (B) among included studies. Combined RR was calculated by a random mode.

Using the methods described above, the OS and/or DFS of 849 patients in 8 studies were analyzed. The main results of this meta-analysis are shown in Figure 2. Five-year OS rate was extracted from 5 studies. The meta-analysis of the 5 studies for the prognostic value of p16^INK4a^ overexpression showed that p16^INK4a^ overexpression was associated with a favorable OS. This was obtained from the DerSimonian–Laird random-effects model with a value of 0.42 (95% CI: 0.24–0.72, P = 0.002) (Fig. 3), although there was heterogeneity between studies (I^2^ = 58%, P_h_ = 0.05). In fact, 4 out of 5 studies have also concluded p16^INK4a^ expression as a favorable prognostic factor in cervical cancer patients (Fig. 3A).

### p16^INK4a^ expression and DFS in cervical cancer

Meta-analysis of 4 applicable studies showed that p16^INK4a^ expression was associated with favorable DFS (RR: 0.60, 95% CI: 0.44–0.82, P = 0.001; Fig. 3B). No heterogeneity was observed between these studies (I^2^ = 0%, P_h_ = 0.78).

### Sensitivity analysis

In order to test for a bias introduced by the low numbers of available eligible publications we performed a sensitivity analysis. For this a single study involved in the meta-analysis was omitted for each round of analysis to investigate the influence of the individual data set of the particular study to the pooled ORs. We found that the corresponding pooled ORs were not essentially altered by substraction of any study (data not shown), indicating that our results were statistically robust.

### Publication Bias

Begg’s funnel and Egger’s test were performed to assess the publication bias in this meta-analysis. The shape of the Funnel plots did not reveal obvious evidence of asymmetry. Egger’s test showed no significant publication bias for tumor grade (P = 0.18), TMN classification (P = 0.835), tumor size (P = 0.851), lymph node status (P = 0.051), vascular invasion (P = 0.142), DFS (P = 0.602) and OS (P = 0.624) (Table 2).

**Table 2:** Egger’s test of funnel plot asymmetry.

clinicopathological parameters	t value	df	P value
tumor grade	0.59	11	0.18
TMN classification	0.28	10	0.835
tumor size	0.11	5	0.851
lymph node metastasis	1.73	16	0.051
vascular invasion	3.68	4	0.142
disease free survival	0.67	2	0.602
overall survival	0.29	4	0.624

df: deflection.

## Discussion

The p16^INK4a^ protein is a cyclin-dependent kinase inhibitor that regulates the G1/S cell cycle checkpoint by inactivating cyclin D1-CDK4/6 complex activity and thereby enhancing pRb activity and suppressing cell growth [24]. It was shown recently that HPV-transformed cervical cancer cells are dependent on its expression and knock down will lead to reduced proliferation [6]. Many IHC-studies have demonstrated that p16^INK4a^ protein is highly overexpressed in dysplastic epithelial cells of the uterine cervix and that it is associated with HR-HPV infection. At the same time its expression is basal in normal epithelium and benign lesions due to few spontaneous senescent cells [25]. Furthermore, overexpression of p16^INK4a^ appears to correlate with the degree of cervical neoplasia, which may improve the histological diagnosis and hence the management of cervical lesions [26]. Tsoumpou et al [27] performed a meta-analysis of 61 published studies on the correlation of the p16^INK4a^ immunostaining to the degree of cytological or histological abnormality. They reported that the proportion of cervical smears overexpressing p16^INK4a^ increased with the severity of cytological abnormality. In histology only 2% of normal biopsies and 38% of CIN1 showed diffuse staining for p16^INK4a^ compared to 68% of CIN2 and 82% of CIN3 [27]. Thus, p16^INK4a^ is regarded a surrogate biomarker for cervical intraepithelial neoplasia.

Recently, the clinical significance of p16^INK4a^ overexpression in cervical cancer has been reported by many investigators. However, the results of these reports are still conflicting. To address the predictive value of p16^INK4a^ overexpression in cervical cancer, we performed a meta-analysis of the published studies to obtain a more precise estimation of the above stated association. This meta-analysis summarizes all currently available and relevant data on the impact of p16^INK4a^ overexpression on the prognosis of cervical cancer including 1633 cases. Altogether our results using the pooled RR of OS indicate that a low p16^INK4a^ expression indicates a poorer prognosis for patients diagnosed with cervical cancer than p16^INK4a^ overexpression. Our findings were consistent with the theory that HR-HPV is a triggering factor in the development of cervical cancer, but would concomitantly induce a p16^INK4a^ -mediated protection mechanism [28]. The reason is not clear at present but presumably overexpression of p16^INK4a^ can be recognized by the immune system as an antigen of naturally low expressed protein. This p16^INK4a^ protein can eventually initiate an antitumor response in cervical cancer patients [29].

However, the lack of standardized cut-off points for positive expression could have led to underestimation of the true prognostic significance of p16^INK4a^ overexpression. No clear guidelines are available regarding their use in routine practice [30]. Tsoumpou *et al* [27] reviewed 40 publications where 35 used Klaes’ scoring system. The percentage of p16^INK4a^ positivity of high grade squamous intraepithelial lesion varied between 44% and 92%. Thus, a predictive evaluation of the scoring systems to reach a consensus on the threshold of positivity is needed.

Strong p16^INK4a^ expression is a proven useful surrogate marker for tumors with transcriptionally active HR-HPV, which is known to be associated with less genetically altered and less complex tumors that respond better to therapy and have improved outcomes. The positivity for p16^INK4a^ has been proposed as a prognostic marker for a more favorable outcome in head and neck squamous cell carcinoma and in lung cancer [31], [32]. Some authors have suggested that p16^INK4a^ plays a major role not only in suppression of cell division but also in suppression of lymphangiogenesis and lymphatic metastasis [30].

Our analysis is supported by the clinical observations of Riou et al [33] who reported that HPV-negative cervical cancer patients had a significantly higher risk of overall relapse and a higher risk of distant metastatic tumor than HPV-positive patients. Tumors that lack HR-HPV positivity may have a larger number of mutations in genes coding for cell cycle regulating proteins to be transformed, and thus may be more therapy resistant [34]. This is corroborated by the fact that HR-HPV positive carcinoma generally is more susceptible to radiochemotherapy than HPV-negative counterparts [35].

This meta-analysis has certain limitations that should be considered when interpreting the results. First, our results are based on unadjusted estimates. A more precise analysis could be conducted using the original individual data sets that, however, are not available to us. Nevertheless, such an approach would allow for adjustment by other co-variates including age, ethnicity, family history, environmental factors, treatment and lifestyle [36]. Second, heterogeneity is a potential problem when interpreting the results of meta-analyses. We minimized the likelihood of this problem by performing a careful search for published studies using explicit criteria for study inclusion, precise data extraction, and strict data analysis. However, significant heterogeneity between studies existed in some comparisons. The presence of heterogeneity results from differences in many factors, including the age distribution, lifestyle factors and the standard of the IHC technique used particularly concerning the positivity threshold. Third, only published full text studies were included in this meta-analysis. Non-significant or negative findings may not be published or only published as abstract at conferences and could therefore not be evaluated. We included data of 1633 patients currently available in this meta-analysis that should lay the foundation to perform a larger and prospective study.

In conclusion, despite the limitations of this meta-analysis, our study suggests that p16^INK4A^ overexpression is significantly associated with better prognosis in terms of longer DFS and OS in patients with cervical cancer. Hopefully this analysis will stimulate further research with rigid criteria and large study populations to resolve any remaining controversy of the role of p16^INK4A^ expression for the prognosis of patients with cervical cancer.

## Supporting Information

Checklist S1 **PRISMA checklist.**
(DOC)
